# What of It?

**Published:** 1884-01

**Authors:** Geo. E. Blackham


### WHAT OF IT?

GEO. E. BLACKHAM, M. D.

To the disinterested student of human nature the fuss and flutter made over the "Code of Ethics" by the State and National medical soties has much of the comic element. The New York State Medical Society, a chartered institution or corporation, resolved that its members *might* (not must) meet in consultation any physician legally qualified, whether of their own or any other "school." Whereupon the American Medical Association, an unchartered body, without legal status or authority of any kind, refused to admit delegates from the New York State Society, and even went so far as to refuse to admit to its meetings at Cleveland any members who would not sign a pledge to stand by what is known as the "Old Code." After this piece of impertinence on the part of the "Judicial Council," one of the doctors, who had been most prominent in enforcing his strict views of ethics, went out and gave a practical demonstration of their value by engaging in a fight with another local doctor over some question of precedence in the local committee of arrangements.

Now we have in this State (New York) two organizations—one to secure and the other to prevent the readoption of the "Old Code," so that it seems to be looked upon by both sides as a very important matter. But is it so? Let us see. Suppose, what is far from probable, that the "Old Code" is readopted by the Medical Society of the State of New York, what will be the result? Probably nothing at all further than to put on record the fact that bigotry and intolerance still have power in what ought to be the most liberal, tolerant, and progressive of professions.

Of course, if some liberally-minded doctor should persist in asserting his independence, and meeting, let us say, a homœopathic physician in consultation, he might be brought up before his county society, tried and expelled; but it is more than probable that the strong arm of the law would, if he chose to invoke it, restore him to membership and teach the county medical society that it was subject to and not superior to the laws of the State from which it derived its own existence, and that it had no authority to declare irregular, and hence unworthy of association, any physician whom the State law had endorsed as "legally qualified."

But supposing the expelled member did not choose to invoke the aid of the law, or having invoked it, should find it sustaining the action of the county medical society, what then? Would the expelled member suffer in any way in consequence of the said expulsion? Or, in other words, would the expulsion have any punitive effect? If the act of 1823 were still in force, the license of the county medical society would be necessary to the legal practice of his profession, and hence expulsion from the county society, with its natural corollary, the revocation of its license, would deprive him of his legal standing as a physician, and hence of his livelihood. Or, if the provision of the Revised Statutes of 1830, requiring *every* practicing physician to become a member of a county medical society, and punishing failure to do so by forfeiture of license to practice, the same deprivation of livelihood would follow expulsion from the county society. But, as the law now stands, if the expelled member has a diploma from some incorporated medical college or medical school of this State, or endorsed by such incorporated medical school or college, and he shall have duly registered in accordance with the law of May 29, 1880, then his expulsion from the county society does not affect his legal status as a physician at all, but only his personal relations to that society and the members thereof. If a member were to be expelled for any act in itself disgraceful or criminal, there is no doubt but that the effect of such expulsion would be to brand him as an outcast, and thus materially affect his public standing, and consequently diminish his practice; but in the present state of public opinion expulsion for consultation with a legally qualified physician of another school than his own would simply be regarded as an act of bigoted persecution, which would enlist public sympathy on his side and probably prove an admirable advertisement for him. It is thus seen that expulsion from the county society, under these circumstances, would not affect his legal standing as a physician, nor would it be likely to have an injurious effect upon his practice. There remains then only the deprivation of the privilege of attending the meetings of the society, and, nominally, of consulting with its members. As the meetings of most county medical societies are merely social gatherings, with only the faintest pretense of scientific interest, the loss from non-attendance at their meetings would be but slight. In fact, the meagre attendance of those entitled to be present is the best evidence of the slight value set upon the privilege.

Personally, I have never yet seen a meeting of a county society at which there was anything like a full attendance of members, or at which any real contribution to scientific medical knowledge was made by any member; and this I believe to be the general condition of facts of the county medical societies of this State, with perhaps a few honorable exceptions. In the days when medical periodicals were few and mail facilities poor and costly, the county societies may have been of value for the interchange of ideas, opinions, discoveries, new methods of treatment, etc.; but now if a physician has anything of value, or which he thinks is of value, to say to the profession, he usually prefers to seek, through the medium of some medical journal, a wider audience than his county society can afford him. Hence, the best papers go to the journals instead of to the societies, and are thus quite as available to the expelled member after as before his expulsion. There remains then only the question of consultation with such physicians as remain orthodox on the question of ethics. Doubtless this would be, to some extent, operative; but as those who advocate freedom of consultation include many of the most eminent and progressive men in the profession, the expelled member would find himself in excellent company, and his sufferings and inconveniences, arising from the refusa of the orthodox members, would probably not be great.

It appears then, that so far as the individual members of the profession are concerned, it makes very little difference what code of ethics, if any, the State Medical Society and its subordinate county societies adopt, as they have no efficient means of compelling obedience or punishing disobedience. With regard to the State and county societies themselves the case is different. The re-enactment of the old, bigoted, exclusive code, and an attempt at its enforcement, can only result in driving from their ranks their most liberal and independent members—the men from whom the majority of the discoveries and advances in diagnosis, treatment, and pathology may be expected.

So far as the liberal-minded members of the profession are concerned, it is not of the least consequence whether the State and county resolve to permit or forbid consultations with legally qualified practitioners, not within their fold. They have no power to enforce their commands, and their fulminations on the subject belong to the same category as the "Pope's bull against the comet," full of sound and fury, perhaps, but signifying nothing and absolutely of no importance or interest to any person or thing in this wide world except themselves.

If they will get together, stop quarreling over codes of ethics and rules of etiquette, organize for practical scientific work, encourage the presentation, discussion and publication of medical and surgical papers, have their committees report regularly and faithfully upon the advances made in various departments of medicine, labor for the raising of the legal standard of education required for admission into the profession, and extend the right hand of fellowship to all educated and studious medical men, whatever their therapeutic ideas, they can do much for the good of the profession; or by continuing as they are, they can remain ciphers in the great problem of medical science, unwilling to advance and impotent to retard the advance of knowledge and the increase of the dignity, importance and usefule ss of the medical profession.

